# AOTA/NBCOT Occupational Therapy Licensure Compact Initiative Moves Forward

**DOI:** 10.5195/ijt.2019.6296

**Published:** 2019-12-12

**Authors:** Chuck Willmarth, Shaun Conway

**Affiliations:** 1 American Occupational Therapy Association (AOTA), Bethesda, MD, USA; 2 National Board For Certification In Occupational Therapy (NBCOT), Gaithersburg, MD, USA

## CSG CONVENES OCCUPATIONAL THERAPY COMPACT ADVISORY GROUP IN WASHINGTON

The Council of State of Governments (CSG) convened the first meeting of the Occupational Therapy Interstate Licensure Compact Advisory Group from October 29 to 30 at the Hall of the States in Washington, DC. The role of the advisory group is to engage in a thoughtful dialogue regarding the needs of occupational therapists, occupational therapy assistants, students, state licensure boards, and other stakeholders as those needs pertain to the development of an interstate licensure compact.

**Figure:**
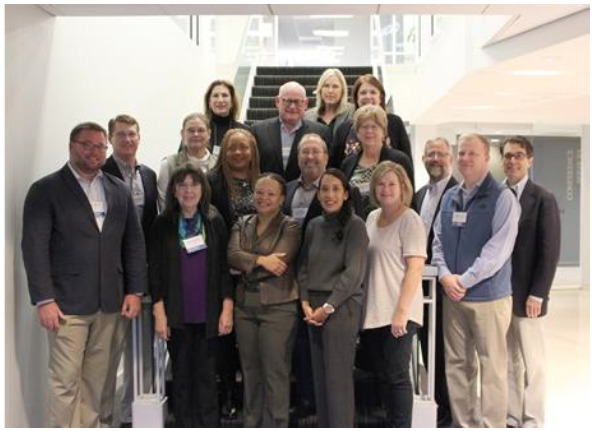
Members of CSG's Occupational Therapy Interstate Compact Advisory Group

The 2-day meeting began with a briefing by CSG staff members to provide the background and history of interstate compacts, and was followed by a review of existing compacts in medicine, nursing, physical therapy, and other health professions. Staff briefed the advisory group on the findings of their research into the state regulation of occupational therapy. A key finding is that the core licensure requirements for occupational therapists and occupational therapy assistants are consistent across jurisdictions. Specifically, all states require OTs and OTAs to graduate from accredited programs, complete supervised fieldwork requirements and pass the entry-level certification exam administered by NBCOT®. The group identified some variations in state requirements; for example, at least 12 states require a state jurisprudence exam (usually an open-book test regarding the state's occupational therapy statutes and regulations).

**While no final decisions were made, the group came to consensus on a number of key points:**

- The licensure compact will be referred to as the “Occupational Therapy Compact” or “OT Compact” for short.
- Licensed occupational therapists and licensed occupational therapy assistants, in good standing, will be eligible to participate in the compact.
- The home state license authorizes practitioner participation in the compact.
- The “mutual recognition model” was recommended by the Advisory Group. This means practitioners must have a home state license and then will apply for privileges to practice in other states.
- A minimum of 10 states will be needed to activate the Compact Commission (a quasi-governmental organization that will implement the compact).
- Practitioners with an encumbered license will not be able access compact privileges.
- Participating states must implement FBI fingerprint-based criminal background checks.

While the Compact Advisory Group addressed a number of key issues, more work is left to be done. CSG has scheduled calls for November and December to continue the dialogue. The group is expected to meet with the administrators of the Physical Therapy Compact and the Nurse Licensure Compact to obtain information about how states have implemented these compacts and the related costs. The group will continue to meet over the next 3 to 6 months. It is expected that the Drafting Team will meet in January or February 2020 to begin drafting the licensure compact language. CSG is developing a communications and stakeholder feedback plan to ensure that the profession and all stakeholders have the opportunity to provide input. Read more about the Interstate Professional Licensing Compact at: https://www.aota.org/Advocacy-Policy/State-Policy/Licensure/Interstate-Professional-Licensing-Compact.aspx

**Figure:**
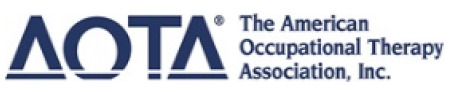
Chuck Willmarth, CAE, is AOTA's Vice President of State Affairs and Health Policy.

**Figure:**
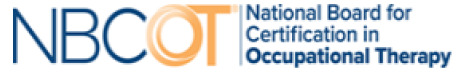
Shaun Conway, OTR, is Senior Director, External & Regulatory Affairs at NBCOT.

Announcement originally appeared Nov. 11, 2019 at https://www.aota.org/Publications-News/AOTANews/2019/Licensure-Compact-Initiative-Advisory-Committee.aspx

and

Nov. 14, 2019 at https://www.nbcot.org/en/News

